# Between two worlds: nationality and identity in Korean immigrants – a qualitative exploration of a sense of belonging in California, the United States

**DOI:** 10.1186/s40359-025-03368-7

**Published:** 2025-09-26

**Authors:** Chulwoo Park, Patricia Angelica

**Affiliations:** 1 Department of Sociology, Anthropology, and Public Health, University of Maryland, Baltimore County, Baltimore, MD 21250 USA; 2 Department of Public Health and Recreation, San José State University, San Jose, CA 95192 USA

**Keywords:** Nationality, Identity, Korean immigrants, Qualitative study, In-depth interview

## Abstract

**Background:**

Korean immigrants are among the top five Asian immigrant groups in California. Many came to the United States with a visa or green card, and later some became U.S. citizens. One’s nationality refers to the country of their legal citizenship, but it is often also the country where they were born. As some people renounce their citizenship from their country of origin, the impact of nationality on identity and the influence of identity on nationality have yet to be studied.

**Methods:**

We conducted a qualitative study using semi-structured, in-depth interviews between March 2023 and July 2023. A total of 24 Korean immigrants in the San Francisco Bay Area, California, participated in this study.

**Results:**

Our findings revealed diverse viewpoints on U.S. citizenship among Korean immigrants. Some expressed a desire to obtain U.S. citizenship, some wished to maintain their Korean citizenship, and a few expressed ambivalence regarding the need for U.S. citizenship. In addition, we found that when citizenship is valued by both individuals and the host country, nationality becomes a more influential factor in shaping the sense of belonging. Participants discussed the connection between nationality and identity, emphasizing recurring feelings of being caught between two identities: Korean and American.

**Conclusion:**

The study suggests that increased access to mental health support and resources is needed to improve the psychological well-being of Korean immigrants. Future research is expected to reach a broader group of Korean immigrants with diverse backgrounds, locations, and experiences to make the findings more generalizable.

**Supplementary Information:**

The online version contains supplementary material available at 10.1186/s40359-025-03368-7.

## Background

### Nationality and identity among Korean immigrants

According to the East-West Center, five states—California, New York, New Jersey, Texas, and Washington—account for more than half of the total South Korean (hereafter, Korean) population in the United States [1], with California alone hosting about half of this population, representing one-fifth of the all Korean populations in the United States [2]. The influx of Korean immigrants to the United States has shaped the nation’s cultural landscape and contributed to diversity [3–5]. A majority of Korean immigrants are naturalized citizens, with many others holding green card holders [4], obtained through family or employer sponsorship [6], contributing to the country’s diverse immigrant demographic.

The U.S. Citizenship and Immigration Services defines citizenship as “a unique bond that unites people around civic ideals and a belief in the rights and freedoms guaranteed by the U.S. Constitution.” Citizenship can be obtained in two ways: naturalization, which is the process of granting U.S. citizenship to a lawful permanent resident, and acquisition of citizenship, which occurs through U.S. citizen parents, either at birth or after birth but before the age of 18 [7]. A lawful permanent resident, an individual who has been legally admitted to the United States, can apply for naturalization based on their permanent resident status after 5 years (or after 3 years for spouses of U.S. citizens) [8].

Korea permits dual nationality but imposes strict regulations. When a child is born in a country that follows the *jus soli* (right of the soil) principle, while their parent is a citizen of a country that follows the *jus sanguinis* (right of blood) principle, the child automatically acquires both citizenships at birth—one from the country of birth and one from the parent’s nationality [9]. For example, a child born in the United States to South Korean parents acquires both U.S. citizenship and South Korean nationality at birth, resulting in dual nationality.

There are three options for those with dual nationality status in Korea. First, an individual can choose to live as a lifelong Korean dual citizen. This requires submitting a “written pledge not to exercise the foreign nationality” before the age of 22. This pledge includes a commitment to fulfill all responsibilities as a South Korean citizen (such as military service for men and taxes) and refraining from using their foreign nationality, such as by not registering as a foreigner or using a foreign passport when entering or leaving Korea. The second option is to renounce South Korean nationality and retain only the foreign nationality. The third option is to renounce the foreign nationality and keep only South Korean nationality [10]. Thus, individuals with dual nationality are generally required to choose one nationality unless they submit a written pledge. However, there are specific exceptions for retaining dual nationality while restoring South Korean nationality. Korean adoptees who were adopted as minors, spouses of Korean citizens, individuals over 65, and those with outstanding achievements may reinstate Korean nationality without giving up their current nationality [11].

The complex interplay between nationality and identity has become increasingly relevant as the Korean immigrant population integrates more into American society. Being an immigrant often involves a balancing act between cultural, social, and personal aspects [12]. Immigrants integrate into the U.S. culture through various elements, including language, education, employment, political engagement, and community participation [13]. For some, acculturating to a new environment is crucial to feeling accepted [14]. However, many face challenges related to legal status and cultural adaptation [15]. Immigrants may find the U.S. immigration system complex as certain visas and green cards, such as employment-based visas, family-based visas, and temporary work visas, require sponsorship [16, 17]. However, the perception of the United States and adaptation to the host country among Korean immigrants, particularly in relation to the concept of nationality and identity, have yet to be studied.

### Purpose

The impact of nationality on identity and belonging, and vice versa, is crucial for understanding how individuals living biculturally navigate their lives. Understanding this dynamic is essential for addressing the challenges faced by those experiencing cultural dislocation, as it can inform policies, social integration programs, and mental health interventions to support immigrant populations in adapting to new environments and forming meaningful connections. Therefore, we conducted this study with the following research question: How do the complexities of nationality and identity influence the formation of a sense of belonging, and how do those factors intersect reciprocally among Korean immigrants in California, United States? Thus, we aimed to determine whether nationality influences identity and conversely, whether identity affects nationality among Korean immigrants in the San Francisco Bay Area, California. By exploring Korean immigrants’ thoughts and beliefs about their nationality, identity, and sense of belonging, the research aimed to provide a nuanced understanding of how they navigate their sense of self and cultural identity within the context of a new cultural environment. Additionally, this study sought to explore valuable insights into the dual aspects of maintaining Korean heritage while embracing American culture, examining how nationality may impact identity and how identity may influence the decision to retain one’s nationality.

### Theoretical framework and contribution

While our study is grounded in established theoretical frameworks, including acculturation theory [18], social identity theory [19], and immigrant transnationalism [20], we refine and extend those frameworks in context to the lived experiences of Korean immigrants. The central argument of the study emphasizes the fluidity of identity, shaped not only by personal experiences but also by cultural expectations from both the host and home countries. By focusing specifically on Korean immigrants, this research provides new perspectives on the negotiation and adaptation of identity within a bicultural context, where the challenges and opportunities of immigration intersect in distinctive ways. This study advances acculturation theory by challenging the prevalent notion that adaptation occurs in a linear trajectory. Instead, it considers identity development as a cyclical, context-sensitive process, in which identity may shift dynamically in response to legal status, perceived belonging, and situational demands—moving beyond the traditional framing of identity as a balance between heritage preservation and assimilation. In relation to social identity theory, which holds that belonging to a stable group offers a constant source of self-definition, this study explores the possibility that identities are often situational and fragmented. It considers how strategic choices to express or suppress aspects of identity—depending on context—complicate conventional ideas of group-based self-definition, indicating that group membership may not always provide a stable or consistent foundation for identity. In addition, this study enhances immigrant transnationalism by acknowledging that it is not merely dual or blended, but rather actively reconfigured and selectively aligned in response to changing social and legal contexts. This perspective highlights a more nuanced, micro-level understanding of transnationalism, suggesting that immigrants may flexibly manage multiple identities rather than consistently embodying a singular transnational identity. Ultimately, this study broadens the theoretical spectrum of immigrant identity construction beyond conventional models by emphasizing identity negotiation as a dynamic, ongoing process that is influenced by both internal and external factors.

### New conceptual insight

We propose *conditional identity alignment* as a preliminary conceptual improvement based on our findings from this qualitative study. This concept describes how immigrants negotiate their legal nationality and cultural identity in practical, situational, and emotional ways. According to our research, Korean immigrants frequently manage a fragmented sense of self, balancing their identity with their nationality—or deliberately maintaining them apart—depending on legal, social, and cultural contexts, rather than seeing identity as binary (Korean vs. American) or merely bicultural. This study contributes to advancing the understanding of fluid identity formation as a dynamic, multi-positional process experienced by immigrant minority populations, with a focus on Korean immigrants residing in the San Francisco Bay Area, California.

## Methods

We conducted a qualitative study through one-on-one in-depth interviews. To collect the demographic information of the participants before an in-depth interview began, we used an online survey called Qualtrics XM (Qualtrics International Inc., Provo, UT, USA). Our research team consists of Asian immigrants who are familiar with the Asian culture. CP, the Principal Investigator and lead author of this study, has led several qualitative research projects on immigrant and minority health. CP is multilingual, including English and Korean, which were instrumental for this study, and has extensive knowledge of Korean immigrant communities in the San Francisco Bay Area, California, having observed their living transitions and sense of belonging. PA, an undergraduate research assistant at the time of the study, participated in designing the interview questionnaire, led a few interviews, transcribed the audio recordings, and analyzed the qualitative data under the supervision of CP.

### Participant selection

There were a total of 24 participants who completed both components, the online survey and the in-depth interview. The inclusion criteria were as follows: (1) participants’ parents must be Korean regardless of their current nationality, (2) must be 18 years old or older, (3) have been regularly attending the church in the San Francisco Bay Area, California, and (4) must be able to complete the online survey and engage in the interview in English. These criteria were introduced at the start of the survey, and only those who met all of them were able to respond to the questions. The age group was selected to ensure that participants were at an age where they could provide meaningful insights into their experiences of transitioning to the United States. Individuals aged 18 years and older are typically in a life stage where they are navigating significant personal and cultural changes, making them particularly relevant to understanding the challenges and adjustments involved in such transitions. This age range also allows for participants to reflect on their experiences more independently, as they are legally adults and may have more autonomy in decision-making.

To recruit participants, we used purposive sampling. There were three phases to identify potential participants through this sampling method. First, we contacted group leaders at a Korean ethnic church, shared the script of the recruitment announcement via text message, and asked them to forward it to their group members. In that message, an online Qualtrics survey link was included to ask their eligibility, willingness to participate in an in-depth interview, demographic information, and email contact information. Those who were qualified and expressed interest in participating in the survey were invited to our study via email. Secondly, we directly reached out to Korean immigrant individuals at church who met inclusion criteria. Third, we continued to ask participants who had completed an in-depth interview to refer us to eligible participants in their networks, and some participants were recruited through a snowball sampling. We did not collect the name of the church they attend, so some participants recruited through word of mouth could be from several different churches. All participants recruited in any of those phases completed the Qualtrics survey before beginning an in-depth interview (as shown in Tables 1 and 2).

**Table 1:** Demographic information of study participants

Total participants of the study, *N* = 24					
Gender, *n* (%)			Highest level of education, *n* (%)		
Male	12	50%	8th grade or less	0	0%
Female	12	50%	9th to 12th grade	0	0%
**Age**,** n (%)**			High school graduate	1	4.17%
20–24	1	4.17%	Post-high school vocational program	0	0%
25–29	2	8.33%	Some college but no degree	0	0%
30–34	9	37.5%	Two-year associate degree	1	4.17%
35–39	6	25%	Four-year bachelor’s degree	8	33.33%
40 and older	6	25%	Some postgraduate school but no degree	1	4.17%
**Marital status**,** n (%)**			Master’s/doctorate/medical/law degree	12	50%
Single/never been married	20	83.33%	No response	1	4.17%
Married	3	12.5%	**Place of employment**,** n (%)**		
Separated/divorced/widowed	1	4.17%	Local, state, or federal government	1	4.17%
**Number of children**,** n (%)**			A for-profit private company, business, or individual	19	79.17%
No child	22	91.67%	Self-employed	1	4.17%
1 child	1	4.17%	Not currently employed	1	4.17%
2 children	0	0%	School, or any educational institution	1	4.17%
3 children	1	4.17%	No response	1	4.17%
**Number of people living in the household**,** n (%)**			**Legal status when first coming to the U.S.**,** n (%)**		
One	11	45.83%	Green card (permanent resident (PR))	2	8.33%
Two	9	37.5%	Born with U.S. citizenship	2	8.33%
Three or more	4	16.67%	F-1 visa (student)	12	50%
**Area of living**,** n (%)**			F-2 visa (spouses/children of F-1)	1	4.17%
A suburb near a large city	16	66.67%	F-4 visa (sponsored by a U.S. citizen/PR)	1	4.17%
A small city or town	4	16.67%	B-1/B-2 (business/tourism visitor)	2	8.33%
A large city	3	12.5%	J-1 visa (exchange visitor)	4	16.67%
No response	1	4.17%	**Current legal status**,** n (%)**		
**Total household income in 2022 (before taxes)**,** n (%)**			Green card (permanent resident (PR))	7	29.17%
$35,001 - $50,000	2	8.33%	U.S. citizenship	10	41.67%
$50,001 - $100,000	3	12.5%	H-1B visa (temporary employment)	4	16.67%
$100,001 - $150,000	2	8.33%	F-1 visa (student)	2	8.33%
$150,001 or more	13	54.17%	O-1 visa (extraordinary ability visa)	1	4.17%
Do not wish to answer	4	16.67%			

**Table 2:** Specific description of each participant

Participant #	Age (years)	Gender	Status when first coming to the U.S.	Current status	Age upon arrival in the U.S. (years)	Duration of stay in the U.S. (years)	Initial reasons for coming to the U.S.
1	42	Female	B-1 or B-2	U.S. citizenship	11	31	Immigrated with parents. To study from elementary school.
2	32	Male	F-1	Green card	15	17	To study from high school.
3	34	Female	Green card	U.S. citizenship	17	17	To receive more support from her mother’s family in the U.S.
4	32	Female	F-1	Green card	19	13	To study at the university.
5	32	Female	U.S. citizenship	U.S. citizenship	was born in the U.S., went back to South Korea, and then returned to the U.S.	11	To study at the university.
6	39	Male	J-1	U.S. citizenship	25	14	To work at the company.
7	35	Male	F-1	Green card	20	15	To study at the university.
8	35	Male	F-1	U.S. citizenship	12	23	Immigrated with parents. To study from middle school.
9	32	Male	F-2	U.S. citizenship	11	21	To study from elementary school.
10	42	Female	B-1 or B-2	Green card	26	16	To work as a medical professional.
11	30	Female	F-1	H1-B	18	12	To study at the university.
12	46	Female	Green card	U.S. citizenship	11	35	Immigrated with parents. Her relatives with a U.S. citizenship invited her family.
13	36	Female	F-1	Green card	19	17	To study at the university.
14	44	Female	J-1	Green card	27	17	To study at the university.
15	33	Female	F-1	F-1	30	3	To study at the university.
16	42	Female	J-1	U.S. citizenship	21	21	To join the work and travel program.
17	33	Male	F-1	U.S. citizenship	10	23	Immigrated with parents. To study from elementary school.
18	35	Male	U.S. citizenship	U.S. citizenship	was born in the U.S.	35	His parents came to the U.S. to study at graduate schools.
19	29	Male	F-1	H1-B	13	16	To study from middle school.
20	35	Male	F-1	F-1	17	13 (and spent 5 years in South Korea)	To study from high school.
21	25	Female	F-1	H1-B	15	10	To attend a volunteer camp.
22	45	Male	F-1	H1-B	29	16	To study at the university.
23	30	Male	J-1	O-1	28	2	To work at the company.
24	20	Male	F-4	Green card	16	4	Immigrated with parents. To study from high school.

In this study, the decision to recruit participants from a church was made based on the high levels of community engagement among Korean immigrants within church settings, which provided an effective way to access a broad spectrum of individuals. More importantly, our original intention was to explore how a strong Christian faith would influence Korean immigrants in managing the challenges associated with their legal status and in supporting their mental well-being. Through subsequent in-depth interviews and qualitative analysis using inductive reasoning, we recognized the significance of examining how nationality and identity intertwine in shaping participants’ lived experiences in the United States. As a result, we reported this study independently of our original approach related to Christian faith, but we used the qualitative data from the same church participants, who were not required to be church members for this topic. The relationship between nationality by law and identity based on race and ethnicity has been recently discussed [21, 22]. Although citizenship in a nation-state serves as a primary identity for most people, it competes with other types of identity, including race, ethnicity, language, religion, and ideology [23]. Using this theoretical foundation, we investigated how nationality and identity interacted, interconnected, or contradicted one another among a specific racial and ethnic group of immigrants. While the participants were Christian, this study itself was not focused on Christianity or religious belief. Instead, our objective was to examine how nationality and identity intersect and impact the experiences of Korean immigrants, independent of their religious affiliation. For this particular study, the church setting was simply a culturally relevant way to reach participants, allowing us to capture authentic voices within the immigrant community without making faith the central topic of analysis.

### Qualitative approach: one-on-one in-depth interviews

Of the 24 participants, 22 completed their interviews in person at church, while the other 2 participated virtually via Zoom (Zoom Video Communications, Inc., San Jose, CA, USA). Instead of gathering collective perspectives through a focus group discussion, we chose the one-on-one in-depth interview method because it allows for deeper individual exploration of each participant’s lived experiences. By using interviews, we could capture detailed, personal narratives that provide a richer understanding of how nationality, identity, and other factors influence individuals during their transitions. Qualitative interviews are particularly well-suited for uncovering the complexities of these experiences, which might not be fully captured through quantitative approaches. This approach is grounded in the belief that human experiences and social realities are subjective and must be understood within their specific cultural and social contexts. The interviews took place between March 2023 and July 2023. The duration of the interviews was kept between 1 h and 1.5 h (average: 59 min). The semi-structured interviews were conducted using an interview questionnaire and a script. Each interview involved an interviewer and a participant. The interviewers were accompanied by an assistant, whose role was to ensure that the interview goes smoothly.

### Data collection and instrument

Audio recordings were conducted in each interview using voice recorders. The recordings were initially transcribed using the Otter.ai transcription software (Otter.ai, Los Altos, CA, USA). The researchers then manually edited the transcriptions for accuracy by listening to each of the entire 24 audio recordings. These verbatim transcriptions were only made accessible to the researchers of this study. As an incentive, all participants received a $10 gift card upon online survey completion and a $30 gift card upon in-depth interview completion. Regarding the interview questionnaire used for this study, we focused on asking about their story of coming to the United States, their perceptions of nationality and identity, and their life in the United States (Supplementary Material 1).

### Data analysis

The research team reviewed all 24 transcripts and summarized participants’ responses to the interview questions relevant to this study. We used Dedoose (Version 9.2.22, SocioCultural Research Consultants, LLC, Los Angeles, CA, USA), a web-based qualitative analysis software, to systematically analyze the findings using both deductive and inductive reasoning approaches. After downloading each of the 24 verbatim transcription files in Word document format from Otter.ai, we imported them into Dedoose. The initial data segments were based on the interview question numbers (Supplementary Material 1). Within each question, main codes and their child codes were created, and related quotes across 24 transcriptions were identified in the final branch of child codes. Themes arose after being iteratively refined through interpretation and comparison of coded excerpts using Dedoose. Deductive reasoning allowed us to apply predefined themes or theoretical frameworks to the data, ensuring alignment with existing knowledge and concepts. Themes from deductive reasoning were at the hypothesized stage, not being finalized. Inductive reasoning, conducted following deductive analysis, enabled us to establish each of those themes emerging organically from participants’ responses, either crystalizing our initial hypothesis or uncovering unanticipated insights. This inductive approach allowed us to explore the topic beyond our original expectations. This combined approach provided a comprehensive understanding of the data, capturing both expected and novel patterns within participants’ experiences. After then, another round of qualitative analysis grouped by themes using Dedoose was conducted. Within each theme, we developed relevant categories from the participants’ narratives. Then, specific codes with corresponding quotes were created to fully reflect each of those categories (Table 3). Each of the codes from each category within each theme was fully explained in this results section, along with a quote representing a common thought among the participants. To ensure the validity of the analyzed results, we had continuous reflexive discussions to address coding discrepancies and then to achieve consensus on thematic-based interpretations. Table 3 displays the coding table, including themes, categories, and codes.

**Table 3:** Coding table: themes, categories, and codes

Themes	Categories	Codes
*Nationality* Some Korean immigrants pursued U.S. citizenship to gain freedom, safety, and legal stability, some others opted to retain their Korean citizenship, cherishing their strong Korean identity, while a few individuals expressed mixed feelings about obtaining U.S. citizenship	Obtaining a U.S. citizenship	Freedom in decision making through U.S. citizenship
		Empowerment and stability through U.S. citizenship
	Maintaining Korean citizenship	Want to keep Korean citizenship because of strong Korean identity
		There’s no difference living with a U.S. citizenship
	Mixed feelings about obtaining U.S. citizenship	Obtaining U.S. citizenship later in life
		Shift from reluctance to acceptance of U.S. citizenship
*Identity* Korean immigrants grappled with the process of shaping their identity as they integrate aspects of their home culture with new experiences they encounter in the host country	Identify as somewhere in between Korean and American	No full understanding of either one of the cultures (Korean or American)
		Strong Korean identity in America but feel more Americanized when in Korea
	Living in the U.S. for the opportunity does not change identity	Positive feelings towards identity as a result of having more opportunities in the U.S.
		Less importance of being American due to the diversity in California
		Temporary employment opportunity in the U.S.
	The approach toward American identity	The substantial gap between Koreans living in the United States and those who have spent their entire lives in Korea
		Accepting American culture into a lifestyle
		The deep love of being American: acceptance and belonging
	Korean culture contributes to a strong Korean identity	Being a minority strengthens their Korean identity
		Will never be American due to strong association with Korean culture
		Strong Korean identity influences personality
*Relationship between nationality and identity* Nationality does not seem to reflect identity, but identity could influence Korean immigrants’ decision to choose their nationality	Nationality does not reflect identity	Nationality does not define who I am
		Legal status does not change Korean identity
	Identity generally reflects nationality but could be separate from it	Identity reflects nationality
		Identity can be separated from nationality

### Ethical consideration

This study received expedited review approval at a university located in the San Francisco Bay Area. Participants were provided consent notices both at the start of the online survey and prior to engaging in the in-depth interviews. Potential risks such as psychological risk and uncomfortable emotions were addressed, and free mental health resources were included in each consent notice. At the beginning of each interview, participants were informed that their personal information would be kept confidential, and they had the right to decline to answer any interview questions. To prevent identification, each participant was assigned a number which was utilized for each quote in the results section.

## Results

Participants were asked about their perception of nationality (citizenship) and identity (culture, values, beliefs, and customs). Their thoughts on nationality and identity were grouped into the following three central themes: (1) nationality, (2) identity, and (3) the relationship between nationality and identity. The Results section is organized according to the coding table (Table 3). Each code was accompanied by a description and quotes that exemplified the common thoughts of sub-groups of individuals. For instance, the responses of participants could reflect their initial legal status upon arrival in the United States, the reasons for their initial arrival, or their current legal status (Table 2). Their diverse perspectives were categorized into common sub-groups of opinions within each theme, and then corresponding subsequent codes were introduced that aligned with one or two quotes that accurately represent their thoughts.

### Nationality

Some Korean immigrants pursued U.S. citizenship to gain freedom, safety, and legal stability, some others opted to retain their Korean citizenship, cherishing their strong Korean identity, while a few individuals expressed mixed feelings about obtaining U.S. citizenship. Each participant had a unique experience as a Korean immigrant living in the United States. Their journeys were shaped by diverse immigration pathways, as they arrived with various types of visas, including student, work, and family-based visas. Over time, many participants experienced one or more changes in their legal status, such as transitioning from temporary visas to permanent residency or eventually obtaining U.S. citizenship. This fluidity in legal status influenced each participant’s views on nationality and citizenship in complex and individualized ways. While some participants felt a strong sense of national belonging to Korea, others found their connection to the United States growing over time. Perspectives on nationality varied widely; for some, citizenship was a practical necessity, while for others, it represented a significant shift in identity and belonging. This diversity highlighted the nuanced ways that legal status, immigration pathways, and personal experiences intersect in shaping immigrants’ views on identity and national affiliation.

#### Obtaining U.S. citizenship

##### Freedom in decision making through U.S. citizenship

Obtaining U.S. citizenship is often seen as a significant milestone for immigrants, marking a transition from temporary status to permanent membership within American society, with multiple benefits such as immunity from deportation, access to federal benefit programs, and the ability to sponsor family members for green cards. It also enables privileges such as voting, running for elected office, traveling without visa restrictions to over 180 countries, and holding federal government jobs [24]. For many, the journey to citizenship was not only a legal process but also an emotional and identity-shaping experience. It represents both a sense of security and a recognition of belonging, as it grants immigrants access to rights and privileges that are unavailable to non-citizens. A few participants highlighted that being a U.S. citizen relieves concerns about status and limitations on personal choices. Participant #16 additionally mentioned that retaining Korean nationality would impose constraints on her decision-making. She valued the autonomy and flexibility that U.S. citizenship provides, enabling her to make decisions based on personal preferences:

I think it [(U.S. citizenship)] gave [me] more freedom. If my nationality [were] still Korean, I would have to worry about [my] status, and there might be certain things I cannot even choose. I cannot even think about it [(what I wish to do)]. But at least now I can freely think and decide on my own. (Participant #16)

##### Empowerment and stability through U.S. citizenship

However, the path to citizenship can be long and uncertain, often requiring years of paperwork, interviews, and legal hurdles. Participant #16’s experience reflected the unique pressures that international students face, especially in balancing academic responsibilities with the uncertainties of maintaining legal status. The need to repeatedly secure work authorization added an ongoing layer of stress, contrasting with the stability she now associates with U.S. citizenship. This experience highlighted the mental and emotional strain tied to temporary legal statuses, which often influence immigrant students’ perspectives on security and belonging in the United States:

When I was in college and my status was a student, I was driving and then I see [the] homeless was begging for money. And then I was thinking, ‘Oh, at least you’re a citizen!’ (*laugh*) [Before getting U.S. citizenship, ] I had to struggle working permit thing because every semester, I ha[d] to renew it, it’s kind of stress because it’s not okay. (Participant #16)

#### Maintaining Korean citizenship

##### Want to keep Korean citizenship because of their strong Korean identity

For some individuals, a deep connection to their cultural heritage drives the desire to preserve their nationality or citizenship. Many participants identified strongly with their Korean roots. However, while they acknowledged the influence of American culture on their perspectives and values, their sense of self remained firmly tied to their Korean identity. Participant #11, for instance, reflected on how American culture had shaped her worldview, but she expressed contentment with her Korean identity and did not feel compelled to pursue American citizenship:

The way I see the world and interact with people, and the way I have built my point of view on values, have been heavily influenced by American culture. But I still hold my identity as Korean. In fact, I don’t know if I want to get American citizenship. I would be happy without it. (Participant #11)

Similarly, Participant #14 expressed a strong attachment to Korea and her Korean identity, which influenced her decision to retain Korean citizenship:

I still love Korea and I love to be a Korean as well. That’s why I just don’t want to get the [U.S.] citizenship. (Participant #14)

The length of participants’ residence in the United States varied, ranging from 2 to 31 years (Table 2). However, as seen with Participant #11 (12 years) and Participant #14 (17 years), a longer stay in the U.S. did not necessarily lead to a shift in their citizenship preferences, highlighting the enduring importance of their Korean identity.

##### There’s no difference in living with a U.S. Citizenship

Individuals with H-1B visas are permitted temporary employment in the United States [25]. At the time of the interview, a few of the study participants (4/24, 16.67%) held H-1B visas (Table 1). Participant #22 expressed a clear preference for maintaining his Korean citizenship while planning to remain in the United States as a permanent resident. This participant shared that he did not feel the need to engage in U.S. civic activities, prioritizing his Korean nationality over involvement in the American political system. He reflected on his stance as follows:

I’d like to keep my Korean citizenship. Just staying as a permanent resident. I don’t think that makes any difference to U.S. politics. Or I am not super interested in contributing or volunteering to the U.S. society. (Participant #22)

#### Mixed feelings about obtaining U.S. citizenship

##### Obtaining U.S. citizenship later in life

Better living conditions and better education are among the common reasons for immigration to the United States [26]. However, Participant #20 preferred to retain his Korean citizenship for a period of time. He initially did not consider acquiring U.S. citizenship, as he valued his Korean citizenship. He shared that his focus was on his work rather than citizenship at that time:

I never thought about getting U.S. citizenship. As long as I can work here [(in the United States)], I want to be a Korean citizen for a while. (Participant #20)

Later in his interview, Participant #20 explained that his decision to pursue U.S. citizenship in the future was motivated by a desire to provide a better environment and education for his future family. He saw the United States as offering opportunities that could benefit them:

The U.S. has much better processes and educational backgrounds…. So the motivation is so that my future family can get a better environment and better education. (Participant #20)

##### Shift from reluctance to acceptance of U.S. Citizenship

Many participants (9/24, 37.5%) initially arrived in the United States with a visa or green card but later became U.S. citizens. For some, the decision to apply for citizenship was not immediate and came after a period of reflection. Participant #3, who came with a green card, explained how she initially resisted applying for U.S. citizenship. At the time, she did not see a significant difference between holding a green card and being a U.S. citizen:

I didn’t want to be a [U.S.] citizen because I felt no different living with a green card. I still need to pay taxes. So in my life, there’s nothing affected, whether I have a citizenship or green card. So I told her [my mother], that I don’t want to apply for [U.S.] citizenship. (Participant #3)

Possessing U.S. citizenship entails the privilege of enjoying rights and freedom [27]. Participant #3 had been reluctant to pursue citizenship, but over time, she recognized the advantages it offered. After a few years, she changed her perspective and decided to obtain U.S. citizenship, ultimately holding dual citizenship. She then emphasized the benefits of U.S. citizenship, such as the increased freedom and sense of safety it provided:

I think [U.S. citizenship] gave me more freedom to go anywhere without having any time limitation…. I also have a feeling that if something happened to me in another country, I know that the U.S. government will protect me, assuring me that I’ll be safe. (Participant #3)

### Identity

Korean immigrants grappled with the process of shaping their identity as they integrated aspects of their home culture with new experiences they encountered in the host country. Identity plays a crucial role among immigrant populations, serving as a bridge to their cultural roots and providing a sense of stability and comfort in a new, often unfamiliar environment. For immigrants, identity can foster a vital feeling of belonging, helping them navigate the challenges of adjusting to a different culture while maintaining connections to their heritage. In this study, participants were asked about their sense of identity, and their responses highlighted a range of perspectives. Some participants expressed a strong attachment to their Korean identity, emphasizing the importance of maintaining cultural traditions, language, and values. Others felt they had integrated into American culture to the point where they identified as American, seeing this identity as a reflection of their social integration and lived experiences in the United States. Still, others described their identity as existing somewhere between Korean and American, acknowledging influences from both cultures without feeling fully aligned with one or the other. This in-between identity illustrates the complex, evolving nature of self-perception among immigrants, who often balance their sense of heritage with the realities of their new cultural context.

#### Identity as somewhere in between Korean and American

##### No full understanding of either one of the cultures (Korean or American)

Immigrants may find it difficult to navigate between two cultures [28]. When asked whether they consider themselves with a strong Korean identity or more Americanized, most participants responded “50/50” or “in-between.” Participant #12’s response highlighted the reason behind the “in-between” is due to the difficulty in understanding both Korean and American cultures:

I think I’m half, 50/50. Sometimes I can’t understand Korean people, but sometimes I can’t understand American culture either. (Participant #12)

##### Strong Korean identity in America but feel more Americanized when in Korea

Some participants become Americanized without noticing it and did not realize this until they visited Korea, after living in the United States for a long period of time and being continuously exposure to American culture. They experienced a blending of their Korean identity with American influences, resulting in an evolving sense of self that reflects aspects of both cultures. A number of participants expressed a strong attachment to their Korean heritage while also acknowledging the significant impact that living in the United States had on their personal identity. This blending of identities often became more apparent when they returned to Korea after extended time in the United States:

I am Korean…. I value and live in a Korean way, but at the same time, when I visited Korea last year, when I met my friend, I actually also felt like I have changed a lot. I am more Americanized now. (Participant #10)

In addition to the blending of identities, feeling caught in between two identities was frequently mentioned among participants, as they struggled to fully connect with either their ethnic Korean identity or their adopted American identity. This tension arose from the complexities of straddling two cultures—Korean and American—while also grappling with the reality of their citizenship status. A few described the difficulty of connecting with Korean friends who may perceive them as “too Americanized.” On the other hand, when interacting with Americans, they were often seen as a foreigner despite the years lived in the United States. The in-between identity has created a barrier when communicating with people from the two identities and led to a lack of sense of belonging:

I am in between. When I talk to my Korean friends, I’m too Americanized so they don’t understand my concerns or worries. When I talk to Americans, they see me as a foreigner…. So I felt like I can’t completely open up to them because there is always a mismatch and gap between either group. (Participant #5)

#### Living in the United States for the opportunity does not change identity

##### Positive feelings toward having more opportunities in the United States

Educational opportunities are a primary reason that many immigrants came to the United States [26]. Similarly, for many participants, the decision to coming to the United States had been motivated by the desire to access a broader range of academic prospects. Half of the participants (12/24, 50%) had initially arrived in the United States on F-1 or student visas. These individuals expressed appreciation for the diverse educational paths available in the United States, which they felt were more flexible and expansive compared to the opportunities in their home countries. Participant #1 reflected on how the educational environment in the United States had provided a greater sense of freedom and exploration:

It’s a wonderful thing because it gives us an opportunity to be exposed to a variety of educational opportunities and freedom. Whereas Korea is somewhat limited. (Participant #1)

##### Less importance of being American due to the diversity in California

California is one of the top 10 states with the most racial diversity, with Asians being in the top 3 racial groups in the state [29]. However, some participants have also lived in other states with fewer Asian residents. They noted differences in how American identity was perceived across various regions of the United States. Participant #22 shared his observations from his time in North Carolina and Texas, where he felt that American identity held more significance compared to his experience in areas with more racial diversity:

There were many non-American people and a lot of H1B temporary workers [at my workplace]. All pursue green cards and permanent residency, but I don’t think that being an American is important or matters much. But when I visited North Carolina or Texas, I felt that being an American is quite important to them. (Participant #22)

##### Temporary employment opportunities in the United States

Aside from educational opportunities, many immigrants came to the United States for employment opportunities [26]. Several participants stated that the United States is a foreign country, emphasizing that their primary connection lies with their Korean background, while the United States is simply a foreign space. This view highlighted the idea that, despite living in the United States, the cultural and emotional ties to Korea remained central. For some, the United States was seen more as a place of work rather than a home. Although currently residing in the United States, Participant #23 viewed his time in the United States as more of a business trip due to the professional opportunities provided:

I miss speaking in Korean and working in Korean. When you work for tech companies, you have more opportunities in the U.S. Living here is like my work, business trip. So I don’t feel anything [even] if something weird happened. I feel like [the U.S. is] a foreign country. (Participant #23)

#### The approach toward American identity

##### The substantial gap between Koreans living in the United States and those who have spent their entire lives in Korea

Some participants strongly identified as Korean, while others saw themselves American. Reflecting on cultural identities, some expressed the belief that they were not fully immersed in Korean culture. Despite efforts to reconnect with their Korean roots, they noticed a distinct cultural gap when conversing with individuals who had lived in Korea their entire lives. For some, this realization led to the conclusion that they could not fully identify as culturally Korean. Participant #8 concluded that, although he acknowledged his Korean heritage, he saw himself as fully American:

I’m not fully culturally Korean. I don’t keep in touch with Korean drama most of the time…. Nowadays, I do try to watch Korean news. But I went back to Korea, and when I talked to people who have lived in Korea for their whole lives, it’s like there’s a substantial gap between them and me. So I’m not fully Korean…. I consider myself fully American, but I have Korean heritage. (Participant #8)

##### Accepting American culture into a lifestyle

Participant #24 shared his approach on how he navigates cultural integration in the United States. He emphasized that while he respected and was open to understanding American culture, he did not view himself as fully adopting it. Instead, he approached cultural aspects as something he could incorporate into his lifestyle without necessarily identifying with them. This reflected his willingness to accept the cultural beliefs of others while maintaining a distinction between acceptance and personal adoption. He clarified that while he was open-minded, he drew a line when it came to conforming to beliefs or behaviors that did not align with his own values:

I’m just accepting their culture into my lifestyle. Not that I identify as American. It’s just what their culture believes. I said, ‘Sure, you can believe it? I’ll accept your beliefs,’ but not to a certain point where I will behave what they believe is right. (Participant #24)

##### The deep love of being American: acceptance and belonging

In addition to the “substantial gap” between Participant #8 and those who have lived in Korea for their entire lives, he identified himself as American because he believes that most people in the United States would consider him American. He expressed a deep love for the United States and a sense of loyalty to the country. He stated that the United States is “my country,” underscoring a strong connection to the country. Participant #8 emphasized his day-to-day interactions that suggest a positive and inclusive experience, feeling recognized and accepted as an American:

I fully identify as an American and I love this country. This is where my loyalty is, this is my country…. I also feel like at this time, the majority of America would accept me as an American. The people I interact with on a day-to-day basis would consider me as fully American. (Participant #8)

#### Korean culture contributes to a strong Korean identity

##### Being a minority strengthens their Korean identity

Some of the study participants spent time living outside California. Participant #5 reflected on her experience as a minority in Ohio, where the Asian population is not prominent [30]. She mentioned that she faced discrimination and racial slurs in this predominantly white state. Studies have found that racial discrimination can be a threat to one’s identity and racial microaggressions may have a long-term effect on their psychological well-being [31, 32]. The most common negative outcomes of racism and discrimination are stress and anxiety [32]. However, she showed a different perspective on how her experience of discrimination led her to be more aware of her minority status and Korean identity. Despite the challenges, she was able to channel the negativity into a source of pride for her Korean heritage and broader Asian identity:

Living as a minority in Ohio, I’ve been discriminated against. People called me with racial slurs. That made me more alert to my minority-ness, my Korean-ness. I transfer that into being proud to be Korean or an Asian in general. (Participant #5)

Another participant experienced discrimination during her high school years, but she was pleasantly surprised by the cultural environment when she began working full-time. Being part of a team with a strong Asian influence strengthened her sense of identity as an Asian. Surrounded by colleagues who shared similar cultural values, she found a newfound sense of belonging and comfort that contrasted sharply with her high school experiences. This positive environment allowed her to connect more deeply with her heritage and feel more confident in expressing her cultural background. The supportive atmosphere at work not only reinforced her identity but also helped her see the value of diversity in a professional setting:

I’m in my boarding high school…. Most of the students were rich White students in the local area. I would mostly hang out with the boarders who are also Asians. When we would just pass by the group of white students, [they yelled at us], ‘Go back to your country!’…. Now I’m [in] my first full time job…. I’m so shocked [that] I’m always surrounded by Chinese people, even though it’s an American company. You have to follow [and] obey your manager…. Then it could be very non-American working style. (Participant #21)

##### Will never be American due to strong association with Korean culture

Most participants (22/24, 91.67%) were born in Korea and grew up in Korea before immigrating to the United States. Although most participants came to the United States at a young age, they often described a strong cultural identity tied to their upbringing, including language, food, TV shows, and family traditions. This deep connection to their Korean roots often shaped their sense of self and belonging. Despite living in the United States for many years, some participants felt that they could never fully embrace an American identity. Participant #13, reflecting on the profound influence of her Korean background, shared her perspective on this challenge:

My whole family is in Korea. They all speak Korean. Korean is my first language and I grew up there up until I was fully adult. I left when I was 18 after finishing one year of college. So I can never be American. I’m just… the language, the culture, the TV show, food, everything, family. (Participant #13)

##### Strong Korean identity influences personality

Identity has an indirect impact on attitudes and behavior [33]. Several participants shared their felt that identity was rooted in innate traits that were core to their sense of self. For example, Participant #21 described a strong connection to Korean culture and values, attributing this to her strong Korean identity. This connection often guided her behaviors, as she found Korean cultural traits more in tune with her personality than American cultural norms. This alignment fostered a preference for settings that reflected Korean customs and values, as these resonated more deeply with her personality and social outlook:

I am more comfortable in Korean culture and an environment where there are a lot of Koreans. Also, cultural reasons and my personality in general. It has more Korean traits than Americans, like being polite to the elderly. (Participant #21)

### Relationship between nationality and identity

Nationality does not seem to reflect identity, but identity could influence Korean immigrants’ decision to choose their nationality. The relationship between nationality and identity among immigrants was complex, shaped by cultural background, personal experiences, and adaptation within a new society. For many, nationality represented more than legal status—it was a connection to heritage and belonging. Immigrants often balanced the traditions and values of their home country with those of their new environment, a process influenced by experiences of acceptance or exclusion. Some immigrants initially identified strongly with their country of origin, while others adopted a more bicultural identity over time, blending elements from both cultures. This balance between nationality and identity often shifted, reflecting the evolving nature of belonging as immigrants navigated their lives in a new cultural landscape. We asked two questions: “Do you think your dominant identity (as Korean or as American) should reflect, demonstrate, or match your nationality? Why?” and, conversely, “Do you think your nationality should reflect (demonstrate, or match) your dominant identity? Why?**”** A few participants mentioned that they had not given much thought to the relationship between nationality and identity. These participants expressed uncertainty or indifference on the topic, often stating that they had not previously considered how aspects of nationality and identity might intersect or influence one another. For instance, Participant #19 noted, “I don’t know. I’ve not thought about that. Uh, no opinion, no clue.” The majority of responses, however, were summarized into two categories: (1) nationality does not always reflect identity and (2) identity generally reflects nationality.

#### Nationality does not always reflect identity

##### Nationality does not define who I am "Nationality does not define who I am""?

Nearly half of the total study participants (11/24, 45.83%) held an American passport, yet their reflections on identity revealed a complex relationship between nationality and self-perception. Some participants indicated that while legal status was significant, it did not wholly capture who they were as individuals. Instead, they viewed identity as a deeper and more personal matter, shaped by lived experiences, cultural exposure, and personal growth rather than by citizenship alone. This perspective underscored how identity transcended legal definitions, rooting itself in one’s unique journey and evolving sense of self. Participant #9 emphasized the importance of personal development, suggesting that identity cannot be defined simply by passport:

I think the experiences and the places that I lived throughout my life shaped me as an individual. That’s who I am. My passport is now an American passport. But nationality matters less than who I think I am as an individual. (Participant #9)

##### Legal status does not change Korean identity

Despite living in the United States for years or even decades, many participants still held strong Korean identity that shared their values, traditions, and sense of self. Several participants emphasized that legal identity and personal identity do not always align. This belief underscores the idea that immigrants can embody multiple identities and social statuses, each with its own significance. As they navigated American society, these individuals continually negotiated and integrated aspects of both identities, reflecting the complexity and fluidity of their sense of self:

The way I see [it], identity—or the legal paper—doesn’t necessarily directly translate to who I am. So I can have multiple identities. I can have multiple social statuses. But then my identity as a Korean doesn’t change. (Participant #11)

#### Identity generally reflects nationality but could be separate from it

##### Identity reflects nationality

Being a citizen comes with responsibilities such as defending the U.S. Constitution and nation, paying taxes, serving on juries when assigned, staying informed regarding community issues, engaging in local community activities, and participating in democratic processes including voting. With the listed responsibilities, all American citizens are legally required to abide by them [27]. Overall, participants thought identity reflects nationally. Participant #18 suggested that the act of helping the country is considered an extension of citizenship, implying that citizens actively contribute to the betterment of their nation as an expression of their shared identity and commitment. For many, fulfilling these responsibilities reinforced the link between their identity and nationality, deepening the connection between who they are and the country they call home:

If you’re a citizen, you’re invested in the well-being of the country. Their problems are your problems so you do what you can to help the country. In that sense, you identify with that country. (Participant #18)

##### Identity can be separated from nationality

However, Participant #21 expressed a different perspective, believing that identity does not necessarily have to influence nationality. She valued her U.S. citizenship primarily for the legal security it provided, especially in terms of feeling comfortable working in the United States. In her view, nationality and identity could be held separately, allowing individuals to choose whichever legal status serves them best without compromising their personal or cultural identity. This approach highlighted an alternative way of reconciling the demands of citizenship with personal identity, where legal status serves as a practical tool rather than a core component of self-definition:

I think the legal status… you should just choose legal status whatever [legal] status works for him or her, but can still keep identity separately from nationality. (Participant #21)

## Discussion

We highlighted our approach, which would make a new contribution to immigrant and minority health among Korean populations in the United States. First, this study made contributions to understanding the specific experiences of Korean immigrant populations, which had been underexplored in comparison to other immigrant groups. Second, we analyzed the complex relationship between Korean nationality and U.S. nationality, shedding light on the nuanced ways in which individuals navigated these two different nationalities. Third, we explored how cultural identity and marginalization intersected in this group, providing new insights into their unique challenges. Finally, our research explored the complexities between nationality and identity, offering a fresh perspective on how these concepts were interrelated for Korean immigrants in the United States.

### Significance for Korean immigrant populations

Focusing on Korean immigrants is significant because it adds a unique perspective to the broader discourse on citizenship conceptualization by focusing on a specific immigrant group with distinct cultural, historical, and socio-political contexts. This study explored the intersection of identity, legal status, and belonging, shedding light on how these factors shape their integration into U.S. society, which can inform policies and support systems. The study’s relevance lies in its potential to inform both academic research and practical interventions for immigrant communities. The justification for selecting Korean immigrants in the United States stems from their unique migration patterns, cultural heritage, and experiences that differ from other immigrant groups. Korean immigrants, particularly in the United States, face distinct challenges related to identity and nationality due to their migration patterns and the racialized experiences they often encounter. Unlike some other immigrant groups, Korean immigrants frequently navigate both strong ties to their heritage and pressures to assimilate into American culture. Understanding these dual dynamics offers valuable insights into broader immigrant experiences, making the study of Korean immigrants a critical contribution to the field.

### Korean nationality vs. U.S. nationality

The desire for U.S. citizenship was driven by various benefits, such as avoiding deportation, accessing federal benefits, voting, applying for government jobs, sponsoring family members, and traveling with a powerful passport [24]. For some, it provided legal security and autonomy. Many valued the opportunities and freedoms U.S. citizenship offers, aligning with previous research on its advantages [24, 34]. According to the CATO Institute, immigrants come to the United States for economic opportunities, family reunification, and education for their children [35]. Permanent residents can apply for jobs, receive federal benefits, and are protected by U.S. laws [36]. Our findings show that obtaining U.S. citizenship was based on individual preferences, as many participants still wanted to maintain their Korean citizenship. They felt that a renewable green card offered sufficient benefits and more travel freedom than other visas. Some participants expressed ambivalence about obtaining U.S. citizenship but felt they had no choice but to change their citizenship eventually due to family, safety, and rights. Of the 24 participants, 20 (20/24, 83.33%) had arrived on visas, and 6 (6/20, 30%) of those were now U.S. citizens. We reported that some participants wanted to retain their Korean citizenship due to their strong Korean identity, even if they had been living in the United States for a long time. However, we did not ask participants with permanent residency how many years had passed since they received their green card, or how their thoughts about obtaining U.S. citizenship might change as the number of years as a permanent resident increase. Some participants may have lived as students on F-1 visas for an extended period or relied on H-1B visas for several years, which might have made it difficult for them to begin thinking about becoming U.S. citizens, as they had not yet been naturalized. Future studies could explore the association between the length of time in the United States after becoming a permanent resident and attitudes toward U.S. citizenship.

### Cultural identity and marginalization

The American Psychological Association noted that strong cultural identity enhances social support [37]. Peck and Wagner (2016) explored how cultural norms in Korea and the United States shape individuals’ experiences of belonging and identity, impacting how they view and navigate citizenship [38]. Their findings highlight the importance of intercultural competencies and suggests that embracing intercultural citizenship can foster mutual understanding and respect in increasingly multicultural societies. According to Choi et al. (2001), Korean Americans’ identity is pragmatic and functional, as well as spiritual—an identity that blends the spiritual and moral values of the East with the pragmatism of the West [39]. Participants from Kim’s (2006) study challenged the white entitlement to an ‘American’ identity—they explored ways to dismantle white supremacy, not only through an Asian identity but also through a more broadly politicized ‘minority’ identity [40]. Other discussions on how to break the monopoly of white power focused on politics, as they felt their political invisibility kept them labeled as ‘foreigners’ and ‘non-Americans’ [40]. Similarly, participants in our study emphasized their strong Korean cultural identity, reinforced by being a minority. They felt comfortable in Korean culture, valuing opportunities in the United States without replacing their Korean identity. Of the 20 visa holders, 7 (35%) are now permanent residents. Some expressed a strong desire to maintain their Korean citizenship due to a deep cultural connection. One participant noted that California’s diversity lessened the importance of American identity. Participant’s values, lifestyles, and worldview influenced their decision to retain Korean citizenship.

Marginalization, the feeling of not belonging to any culture, increases the risk of poor mental health and anxiety [41, 42]. Participants’ “in-between identity” reflects this marginalization, contrasting with integration, where one adapts to a new culture while maintaining their native culture [43]. Our study highlighted immigrants’ dynamic perspectives on citizenship and the multifaceted nature of their identity, influenced by cultural background, experiences, and duration of stay in the United States. Many participants felt stuck between two identities, struggling to fully align with either Korean or American communities.

Cohen and Kassan (2018) identified four approaches to cultural identity negotiation of young adult immigrants through the qualitative study: blended, dual, disconnected, and intermediate [44]. Disconnected cultural identity—having no true belonging in both culture of origin and host culture—and intermediate cultural identity—connecting to the values of both cultures but having a greater degree by one culture—support our finding of identity as somewhere in between Korean and American to some extent. Building upon this cultural identity negotiation, we reported that cultural identity may be situational in different locations, with individuals experiencing a stronger sense of identity with their country of origin when residing in the host country, but a greater sense of identity with the host culture when visiting their country of origin.

### Complexities between nationality and identity

This study highlights the intricate connection between nationality and identity among Korean immigrants in the United States. Rather than presenting a binary view of ‘good’ or ‘bad’ immigrants or conveying superficial affirmation, the media shed light on the complex and ambivalent process of immigration [45]. Participants had diverse views on obtaining U.S. citizenship, maintaining Korean citizenship, and the impact on their identity. Liu (2019) reported that while some Chinese migrant wives embraced the entitlements of citizenship, others experienced citizenship differently based on their positions within both private and public spheres. Additionally, some migrant wives actively rejected Taiwanese citizenship, challenging the assumption that all Chinese immigrants aspire to obtain it [46]. Concerns about identity play a role in shaping individuals’ motivation to pursue naturalization. Practical issues (e.g., the cost of naturalization) and identity-related factors (e.g., feeling a sense of belonging in the United States) both influence this motivation [47]. Additionally, greater perceptions of subgroup respect enhance a sense of belonging in the United States, which, in turn, strengthens the motivation to naturalize. Conversely, perceiving low respect can directly reduce motivation to naturalize [47].

### Conditional identity alignment

In addition to acculturation theory, social identity theory, and transnationalism, we examined other relevant frameworks. Segmented assimilation theory has three processes—consonant, dissonant, and selective acculturation—for second generation that is affected by parental human capital and family structure [48], but it may not apply to our study participants because most of them (22 out of 24, 92%) were first generation. Emotional and affective dimensions of multiple belongings that immigrants have [49] may not adequately address our participants’ identity based on situational demands, such as social and legal contexts in the United States. Post-national and hybrid identity constructs broadly encompass multiple intersecting identities from globalization and transnational movements [50, 51], but they lack specificity in explaining the “in-betweenness” of identity struggles that our participants faced in daily life. Racialization and cultural citizenship [52] also potentially miss the micro-level, everyday decisions that participants make based on their specific, situational strategies. Identity negotiation theory emphasizes that immigrants and minorities can create positive identity affirmation through various communication situations [53], but the reciprocal, bidirectional relationship between nationality and identity experienced by our participants is not necessarily related to interactional strategies. Compared to all of the existing frameworks, our primary goal was to revisit the nationality and identity of a specific immigrant minority population, as well as understanding how they are related, interconnected, or contradictory to one another.

Taken together, our results show that identity development is a dynamic, context-dependent reformation rather than only a simple process of acculturation or biculturalism, so we add the new conceptual insight to current theories: *conditional identity alignment*. Some participants described a continuous feeling of being caught between Korean or American identity and expressed difficulty completely connecting with either of those two identities. The relationship between nationality and identity is situational, conditional, and fluid, influenced by an individual’s unique situation, cultural contexts, personal goals, and social and legal status. Instead of being simply bicultural or transnational, it results in a more fragmented and dynamic sense of self. This suggests that conventional acculturation models, social identity theories, and transnationalism frameworks—which typically emphasize linear integration, stable group belonging, and coherent dual identity—may not fully capture the cyclical and multi-positional identities demonstrated by Korean immigrants.

By proposing a preliminary conceptual insight, *conditional identity alignment*, we demonstrated how Korean immigrants selectively activate or suppress specific aspects of their identities in response to their unique social and legal status. Through conditional identity alignment, we contribute to the advancement and complexity of transnationalism and social identity theory by showing that identity is dynamic and often shaped by conscious decisions rather than passive adaptation. For example, depending on the demands of the situation, participants reported feeling culturally Korean but legally American, or vice versa. We argue that, especially in situations where cultural identity and legal nationality do not neatly align, conditional identity alignment provides a useful conceptual refinement for comprehending immigrant identity formation.

### Limitations

We conducted a total of 24 in-depth interviews, which allowed us to reach data saturation, where no new themes or patterns were emerging from additional interviews. However, there were several limitations and potential biases associated with this approach. First, although the goal of this study was not to assess religious belief or faith, the recruitment strategy was centered on identifying participants who attended church on a regular basis in order to improve the effectiveness of purposive sampling. Because it could be possible that the involvement in church and its perspective on community and support from church are critical for participants in establishing the relationship between their nationality and identity as an immigrant, the next study could explicitly investigate how the shared religious affiliation may shape both findings and the generalizability of the study. Second, participants were recruited through purposive sampling, which might lead to a selection bias—those who chose to participate may have stronger opinions or more extreme experiences compared to those who did not. Our inclusion criteria did not include a minimum length of stay in the United States because we hypothesized that individual identity is determined by their personal process of integration into U.S. society, not by how long they have lived in the country. As a result, participants’ duration of staying in the United States varied, ranging from 2 years to 35 years. However, this method may not ensure the reliability and validity of the study. Third, participants in the San Francisco Bay Area may have unique characteristics that influenced the qualitative data. Thus, our findings may not be generalizable to populations in other regions or states. Fourth, the lead author, CP, was personally acquainted with several participants; interviewer bias and confirmation bias may have occurred when CP interviewed a few of them. To avoid these biases, we used a semi-structured interview protocol, and PA, who had no personal relationships with any of the participants, conducted most of the interviews with those whom CP knew. Lastly, social desirability bias may also be a concern, as participants may modify their responses to align with perceived expectations or norms.

### Implications and future studies

The target location, the San Francisco Bay Area in California, is one of the most diverse and densely populated immigrant areas, which may highlight the distinctive characteristics of immigrant communities that are not applicable to less populated immigrant communities in other areas or states. Thus, future research should aim to include a larger and more diverse sample to enhance the generalizability of the findings. By including participants from different demographic backgrounds, geographical regions, and experiences, the next study can better capture the variability in perspectives and ensure that the results are more reflective of the broader population. This approach could also enable the exploration of nuanced differences between subgroups that might be overlooked in a smaller, more homogenous sample. Additionally, we approached the term “identity” broadly, not dissecting it into different elements, such as race, ethnicity, religion, ideology, culture, gender, language, politics, or economy. We used the encompassing term *identity* to refer to both racial/ethnic and cultural identity, Korean versus American in the inner mind, as opposed to nationality identity. The next study can investigate identity by religion, non-religious versus religious, to see whether religious belief could affect any other aspects of immigrants’ identities. Furthermore, future research might explore how the patterns of conditional alignment shape long-term well-being, belonging, and civic engagement among immigrant populations.

## Conclusion

As Korean immigrants feel the need to adjust to the environment and lifestyle in the United States, their psychological well-being needs to be addressed. Access to mental health support and resources can enhance the quality of life for Korean immigrants, helping them navigate the challenges of acculturation, discrimination, and social isolation. Mental health services that are culturally competent and linguistically accessible play a critical role in fostering resilience and promoting mental wellness within this community. This study can be broadened by including a more extensive recruitment strategy for Korean immigrants, ensuring a diverse sample that reflects various socioeconomic backgrounds, regions of origin, and immigration experiences. The findings from such studies are anticipated to be applicable and beneficial to Korean immigrants, informing policies and interventions aimed at improving their mental health outcomes and overall integration into American society.

## Supplementary Information

Below is the link to the electronic supplementary material.

Supplementary Material 1

## Data Availability

The data supporting the findings of this study are not openly available due to participant confidentiality but can be obtained from the corresponding author upon reasonable request.
