# Supplementary material for: Between two worlds: nationality and identity in Korean immigrants – a qualitative exploration of a sense of belonging in California, the United States
Source: BMC Psychol. 2025 Sep 26;13:1029. doi: 10.1186/s40359-025-03368-7 (PMC12465442; doi:10.1186/s40359-025-03368-7)
Supplement: Supplementary file 1 — Supplementary Material 1 [file 40359_2025_3368_MOESM1_ESM.pdf]

In-depth interview questionnaire

Background

Q1: Tell me about your story of when and how you came to the United States.

Q2: How would you see the difference between your nationality and your identity?

- Q2-1. Do you consider yourself with a strong Korean identity, or do you see you have been Americanized? Which identity is more relevant to you (e.g., 50/50?) Can you share your story related to this? What are some specific situations you have felt this way?
- Q2-2. Tell me how you feel about having another (or both) identity as American or Korean with a legal status that allows you stay in the United States?
- Q2-3. How much your nationality as Korean (or American) is important to maintain or strengthen your dominant identity (Korean or American identity)?
- Q2-4. Do you think your dominant identity (as Korean or as American) should reflect (demonstrate, or match) your nationality? Why?
- Q2-5. Do you think your nationality should reflect (demonstrate, or match) your dominant identity? Why?
- Q2-6. Would you want to obtain U.S. citizenship and give up Korean citizenship? Why?

After Coming to the United States

Q3: Have you ever visited your home country, South Korea? How have your impressions and thoughts about South Korea changed since you came here?

Q4: After living in the US for months or years, how do you feel about being here? Have you ever regretted, had mixed feelings, or you're glad to be here?

Q5: How important your family (in Korea, or in the US) is when you stay in the United States? What does family mean to you?
